# Breast reconstruction with deep inferior epigastric perforator flaps

**DOI:** 10.1308/003588412X13373405386457

**Published:** 2012-05

**Authors:** J Cubitt, Z Barber, AA Khan, M Tyler

**Affiliations:** Buckinghamshire Healthcare NHS Trust,UK

**Keywords:** Breast cancer, Breast reconstruction, Deep inferior epigastric perforator flap

## Abstract

**INTRODUCTION:**

Approximately 45,000 women are diagnosed with breast cancer in the UK each year. The success of screening and the introduction of adjuvant therapies have meant that prognosis is improving and an increasing number of patients are seeking reconstruction following mastectomy. The purpose of this study was to evaluate the deep inferior epigastric perforator (DIEP) flap reconstructions performed in Stoke Mandeville Hospital and, through analysis of complications, detail the evolution of the current care pathway.

**METHODS:**

A retrospective analysis was performed of all the DIEP flap reconstructions performed by the senior author (MT) between July 2003 and December 2010.

**RESULTS:**

Overall, 159 flaps were performed on 141 patients (including 36 bilateral flaps). The average patient age was 49 years (range: 28–70 years) and 13% of flaps were risk reducing for BRCA1/2. Twenty-six per cent of patients suffered one or more complication post-operatively, including systemic complications (pulmonary embolism 2%) and flap specific complications (partial flap necrosis 9%, reanastomosis 3%, fat necrosis 9%). Seventy-four per cent had further elective operations including nipple reconstruction (72%), contralateral breast reduction (36%) and scar revision (21%).

**CONCLUSIONS:**

DIEP flaps are a safe and reliable option for breast reconstructions. This series illustrates the significant leaning curve, with complications, operative time and ischaemic time reducing through the series and post-operative haemoglobin increasing. The complications experienced in this series of 159 flaps with no total flap loss provide the framework for the evolution of the current care pathway including pre-operative imaging, peri-operative deep vein thrombosis prophylaxis and analgesia.

Breast cancer is one of the most commonly diagnosed cancers in the UK with more than 48,000 new cases diagnosed in 2009.^1^ The impact of screening and the introduction of adjuvant therapies have meant that the prognosis of breast cancer is improving and an increasing number of patients are now seeking reconstruction following risk reduction and oncological mastectomies. The National Mastectomy and Breast Reconstruction Audit showed that women who had undergone breast reconstruction following mastectomy report higher levels of emotional, physical and sexual wellbeing.^2^ Out of all reconstructions, a delayed reconstruction with an autologous tissue free flap scored highest.

Over the last 15 years, the popularity of perforator flaps and, in particular, the deep inferior epigastric perforator (DIEP) flap has increased because of reduced donor site morbidity when compared with the transverse rectus abdominis myocutaneous (TRAM) flap.^3^ When the DIEP flap was first described by Koshima and Soeda in 1989, there were concerns about a potential high incidence of complications, including flap necrosis, but refinement of operative planning and surgical techniques over the subsequent decade has meant that its morbidity profile is now comparable to the TRAM flap.^4–6^

The purpose of this study was to evaluate the DIEP flap breast reconstructions performed in our hospital over the last seven years and, through analysis of complications, detail the evolution of the current care pathway.

## Methods

A retrospective analysis was conducted of all the DIEP flaps performed by the senior author (MT) since starting DIEP flap breast reconstructions in July 2003 until December 2010. In total, 159 consecutive DIEP flaps were performed in 141 patients with an average age of 49 years (range: 28–70 years). At the time of surgery, all patients had a body mass index of <30kg/m^2^ and were either non-smokers or undergoing treatment for smoking cessation. Patient characteristics are presented in detail in Table 1.

**Table 1:** Patient characteristics

**General**		
	Number of patients	141 (159 flaps)
	Average age	49 (range: 28–70)
**Type of breast reconstruction**		
	Unilateral	123 (77%)
	Bilateral	36 (23%)
	Left	81 (51%)
	Right	78 (49%)
**Timing of breast reconstruction**		
	Immediate	49 (31%)
	Delayed	110 (69%)
**Additional pre-operative therapy**		
	Chemotherapy	86 (54%)
	Radiotherapy	73 (46%)
**Reason for breast reconstruction**		
	Breast cancer	137 (86%)
	Risk reducing	21 (13%)
	Burns	1 (1%)

All patient data were obtained from the medical notes and the electronic theatre management system. Data collection captured pre and post-operative planning, anaesthetic and operative technique, post-operative recovery regimes, complications and further surgery. Statistical analysis was performed with Prism® (GraphPad Software, La Jolla, CA, US).

### Oncological aspects

Eighty-six per cent of DIEP flap reconstructions were for breast cancer. Of these, 80% (110 flaps) were delayed and 20% (27 flaps) were immediate reconstructions. In the immediate reconstruction group, the skin sparing mastectomy was performed either by the referring oncological breast surgeon or, when that was not possible, by the senior author. When indicated, the immediate patients underwent a sentinel lymph node biopsy prior to the mastectomy and the axilla was managed accordingly at the time of the mastectomy. The histological diagnosis in the immediate reconstruction group was ductal carcinoma in situ in 20 patients (75%) and invasive ductal carcinoma in 7 patients (25%). In the delayed group, the mean time following mastectomy was 1.6 years (range: 0.5–14.9 years). The earliest the reconstruction was performed following completion of radiotherapy was at four months.

### Surgical technique

The patients underwent a standard DIEP flap reconstruction but the points highlighted below may differ from unit to unit.

### Pre-operative imaging and venous thromboprophylaxis:

On the day before surgery, all patients undergo pre-operative perforator mapping and measurement of the diameter of internal mammary vessels using duplex ultrasonography. Following the perforator mapping, all patients also receive a prophylactic dose of low molecular weight heparin (LMWH) (dalteparin 5,000 units subcutaneously) and are allowed to go home for the evening to return the next morning for surgery. If a patient is taking tamoxifen, this is stopped two weeks prior to surgery and recommences on leaving hospital.

### Anaesthetic considerations and analgesia:

On the day of surgery, the patient is admitted at 7.30am, marked by the operating surgeon and reviewed by the anaesthetists. Intra-operative monitoring is carried out with an oesophageal duplex probe. There is therefore is no need for a central line or arterial line.

After induction of anaesthesia, an intrapleural block is performed for post-operative analgesia. Once the patient is positioned on the table, the flap edges are infiltrated with a mixture of 1:500,000 adrenaline in Hartmann’s solution. Additionally, bilateral rectus sheath blocks are placed using 40ml of levobupivacaine once the rectus sheath is closed. All patients have an opiate patient controlled analgesia (PCA) system for post-operative analgesia.

### Surgery:

The patient is prepared with an alcoholic chlorhexidine solution and draped. Usually, two surgeons work simultaneously to prepare the internal mammary vessels (trainee) and raise the abdominal flap (MT). An ipsilateral flap is raised, which is rotated by 180º when transferred to the chest so that the inferior portion is superior, allowing the inferior portion to be chamfered to give a smooth medial cleavage and using the thicker umbilical portion to create the fuller lower pole of the breast.

The recipient vessels of choice are the internal mammary vessels, which are approached by resecting a portion of the third rib. The arterial anastomosis is performed with a 9/0 nylon suture and a venous coupler device (Synovis, Birmingham, AL, US) is used for the venous anastomosis. In the majority of procedures, the DIEPs were identified and dissected out completely and no blocks of muscle were taken as a matter of course. However, if two perforators were close to each other, a small amount of intervening muscle was taken between the perforators.

### Flap shaping, insetting and closure:

The majority of the flap shaping is performed once the flap is on the chest and very minimal de-epithelialisation is performed to the flap while on the abdomen. The abdominal wound is closed using a continuous 3/0 PDS® suture (Ethicon, Somerville, NJ, US) starting medially and running laterally, a dermal stapling device and a continuous subcutaneous suture (Monocryl®; Ethicon). The umbilicus is resited using a V-shaped umbilical flap. Closed, active drainage systems are used, with two for the abdomen and one in the breast. Finally, the locations for the pedicle duplex signals are marked on the DIEP skin at the end of the procedure for post-operative flap monitoring.

### Pressure area care:

Throughout the operation, special attention is paid to the patient’s pressure areas: in between the raising of the flap and the anastomosis, the table is tilted from side to side for five minutes at a time; halfway through the procedure, the blood pressure cuff is changed from one leg to the other; and every two hours, the head and heels are massaged and relieved of pressure. This regimen is documented and signed off on the theatre board.

### Post-operative period:

Post-operatively, the patients return to a single room on the ward and receive 1:1 nursing for the first night. The flap is monitored using skin temperature and duplex signals.

## Results

### Peri-operative details

Detailed information on peri-operative details is shown in Table 2. Of the 141 patients, 123 underwent unilateral DIEP flap reconstruction and 18 underwent bilateral reconstruction. Risk reducing mastectomies for BRCA1/2 accounted for 13% of the breast reconstructions.

**Table 2:** Operative details

**Operation times**	**Mean**
Length of operation, unilateral	7.5 hrs (range: 5.5–10 hrs)
Length of operation, bilateral	10.8 hrs (range 8.8–14 hrs)
Flap ischaemic time	86 mins (range: 21–210 mins)
**Microsurgical anastomosis**	**Number of flaps**
Internal mammary	158 (99.4%)
Thoracodorsal	1 (0.6%)
**Venous coupler size**	**Number of flaps**
1.5mm	23 (14%)
2mm	90 (56%)
2.5mm	32 (20%)
3mm	1 (1%)
**Hospital stay**	**Mean**
Length of drains	4.9 days (range: 2–9 days)
Dose of patient controlled analgesia (morphine)	28mg (range: 0–160mg)
Length of admission	6.2 days (range: 3–15 days)

**Figure 1:**
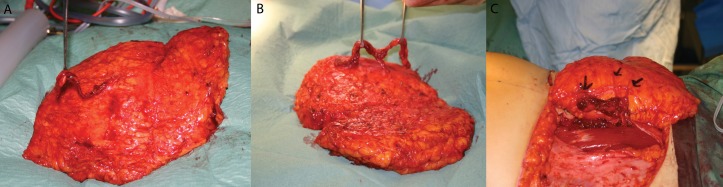
Examples of perforators for different flaps: 1 perforator (A); 2 perforators (B); 3 perforators (C)

The number of perforators ranged from 1 to 6 with a mean of 1.9 (standard deviation [SD]: 0.9). The trend through the series has been to include more perforators with an average of 1.4 in the fist 25 unilateral DIEPs and 2.4 in the most recent 25. (Figure 1 demonstrates several flaps with different number of perforators.) Venous coupler devices were used for 92% of the venous anastomoses.

The mean post-operative haemoglobin level was 9.3 (SD: 1.2g/dl) overall, 9.5 (SD: 1.1g/dl) in the unilaterals and 8.7 (SD: 1.3g/dl) in the bilaterals. The criteria for blood transfusion in our unit is a haemoglobin level of <8g/dl in a clinically compromised or symptomatic patient. Twenty-three patients (16%) needed a post-operative blood transfusion.

### Complications

Post-operative complications were divided into systemic complications and complications specific to the operation. The relevant Clavien–Dindo (CD) complication grade is provided for reference.^7^ The operation specific complications were subdivided by severity into major and minor groups. Thirty-eight patients (26%) suffered one or more complications at some time during their follow-up. Twenty-three patients (15%) were readmitted after discharge, 18 within 30 days (12%). In 28 patients (74%) the complication required surgical management (CD IIIb) and in 10 patients (26%) they were treated conservatively (CD II). The complications are shown in Table 3.

**Table 3:** Complications, number of flaps/patients

	Total	Flaps 1–80	Flaps 81–159	
**Major (number of flaps)**				
	Total flap loss	0 (0%)	0%	0%
	Partial flap loss	14 (9%)	11%	6%
	Reanastomosis	4 (3%)	1%	4%
**Minor (number of flaps)**				
	Infection	14 (9%)	13%	5%
	Wound dehiscence	5 (3%)	0%	6%
	Fat necrosis	15 (9%)	9%	10%
	Haematoma	2 (1%)	3%	0%
**Systemic (number of patients)**				
	Pulmonary embolus	3 (2%)	4%	0%
	Complications due to pressure	2 (3%)	3%	1%
	Blood transfusion	23 (16%)	21%	8%
	Scar revision	34 (21%)	30%	13%
	Implant	7 (4%)	6%	3%
	Latissimus dorsi flap	3 (2%)	3%	1%

In the systemic complication group, the three patients who suffered a pulmonary embolism underwent unilateral DIEP reconstructions and were at the beginning of the series (CD IV). The 2 patients who sustained complications secondary to pressure included 1 scalp alopecia, which resolved spontaneously, in a patient who underwent a 14-hour bilateral DIEP reconstruction that was prolonged due to anastomotic problems (CD I), and 1 suspected compartment syndrome in a lower leg, which required surgical decompression with no muscle necrosis (CD IIIb).

### Additional elective operations

During the follow-up period, 118 patients (74%) underwent an additional elective operation: 114 nipple reconstruction; 34 scar revision; 7 ipsilateral implant; 3 ipsilateral latissimus dorsi pedicled muscle flap for significant partial flap necrosis; 2 abdominal hernia repair and 44 contralateral breast reductions.

### Learning curve

**Figure 2:**
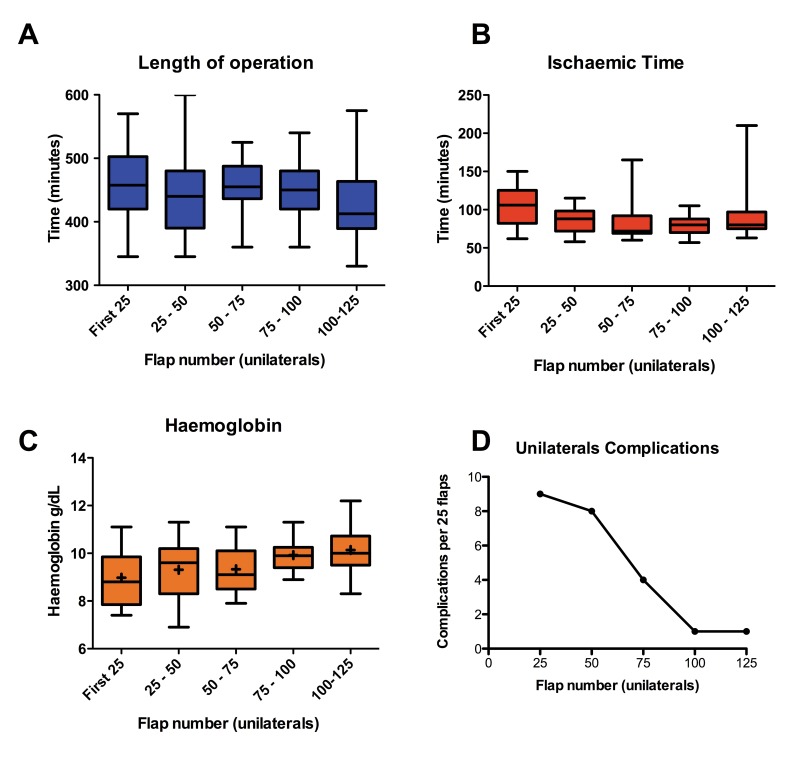
Comparison of sequential groups of unilateral flap repairs: length of operation (A); ischaemic time (B); post-operative haemoglobin (C); complications requiring return to theatre (D)

Univariate analysis or intra and post-operative parameters revealed a significant reduction in length of operation (*p*=0.0062), a significant increase in the post-operative haemoglobin levels (*p*=0.0374) and a reduction in post-operative complications in unilateral DIEP flaps performed later in the series compared with earlier (Fig 2). There was no significant difference between these values in the bilateral DIEP flaps when analysed in three groups with six patients in each group.

## Discussion

Breast reconstruction using autologous tissue has been shown to be a safe and reliable operation throughout the literature.^3,8,9^ The purpose of our study was to evaluate the DIEP breast reconstruction in our institution and to reflect on the evolution of the current care pathway by critically assessing the complications. In addition to pre-operative imaging and overall complications, we chose to focus on areas that, up to now, have been poorly described in the literature: venous thromboprophylaxis, shaping of the flap and post-operative analgesia.

**Figure 3:**
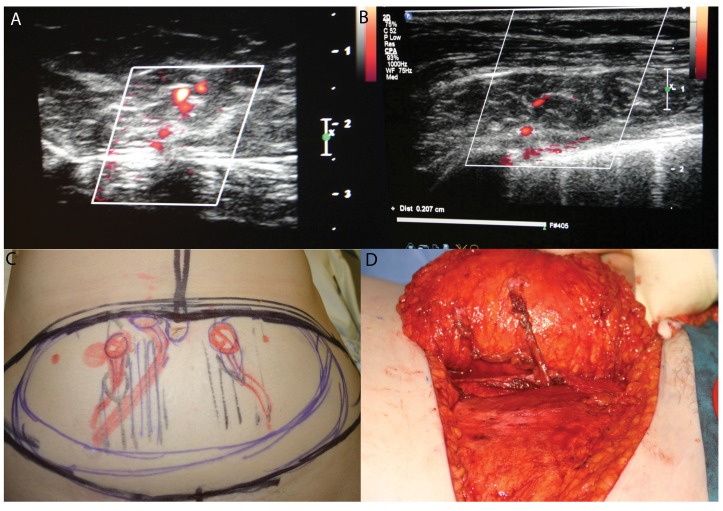
Duplex ultrasonography perforator marking: duplex images (A, B); perforator mapping on the abdomen (C); intra-operative findings (D)

Many publications describe how expensive cross-sectional imaging techniques (computed tomography and magnetic resonance imaging) reduce complications. However, we have described a series of 159 DIEP flaps with no flap loss in which duplex ultrasonography was used pre-operatively.^10,11^ The duplex ultrasonography is performed on the day before the operation by a consultant radiologist with the operating surgeon present. The scan gives real time flow images of the perforating vessels as well as describing the route taken in relation to the muscle.^10^ Figure 3 shows examples of the duplex images and how they correlate with the pre-operative marking and the intra-operative findings. Another advantage of the duplex ultrasonography is that the internal mammary vessels can be assessed for suitability pre-operatively. This is particularly important in identifying large internal mammary veins (3.5mm or larger) when extra care needs to be taken during the approach to the vessels intra-operatively.

Breast cancer, immobility and general anaesthesia are all risk factors for deep vein thrombosis (DVT) and pulmonary embolus. At the beginning of our series, aspirin was used as venous thromboprophylaxis pre-operatively, with some of these patients receiving LMWH post-operatively. However, three patients suffered pulmonary emboli and a regimen was therefore introduced using LMWH.

Patients now receive 5,000 units of subcutaneous LMWH on the night before the operation, none on the day of the operation and 5,000 units again on the morning following the operation, which continues for the duration of their inpatient stay. Patients wear thromboembolic deterrent stockings throughout their hospital stay and DVT prophylactic calf pumps (Flowtron® boots; ArjoHuntleigh, Luton, UK) during the procedure. Furthermore, patients are encouraged to ambulate early. There have not been any subsequent pulmonary emboli since the regimen was changed. In 2011 Lemaine *et al* demonstrated that a similar thromboprophylaxis regimen was safe and successful when used in a microsurgical breast reconstruction population.^12^ In our series, there has not been any increase in the haematoma rate since the introduction of LMWH.

**Figure 4:**
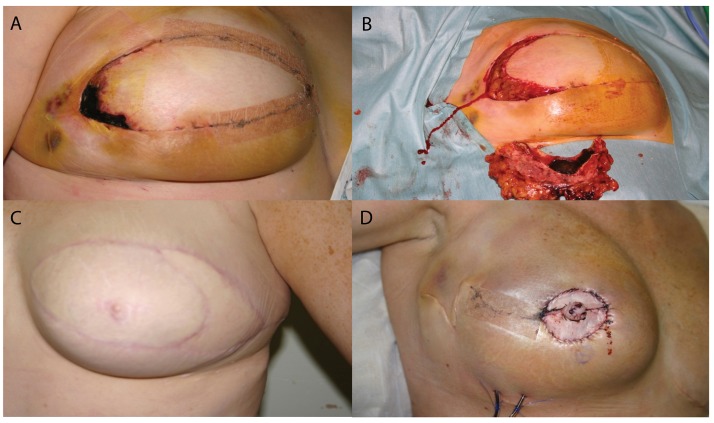
Examples of major complications that required return to theatre: partial flap necrosis in the lateral part of the deep inferior epigastric perforator (DIEP) flap (A); the same DIEP flap after debridement (B); another patient who had significant partial flap loss and needed a pedicled latissimus dorsi flap to replace the volume that was lost (C); venous congestion in a flap that was taken back to theatre and salvaged (D)

The results of our study show that our flap specific complication rates are similar to those reported in major publications (Table 4) and examples of our complications are illustrated in Figure 4. We have demonstrated a distinct learning curve with a reduction in complications as the series progressed, which can be explained by modifications to the operative technique and peri-operative care as experience increases.

**Table 4:** Comparison of outcomes

Authors	Year	Flaps	Total flap loss	Partial flap loss	Fat necrosis
Blondeel^5^ (Belgium)	1999	100	2%	7%	6%
Hamdi^21^ (UK)	1999	50	2%	6%	6%
Hofer^18^ (Netherlands)	2007	159	0.6%	3.3%	7.7%
Chen^19^ (US)	2007	41	0%	0%	12%
Gill^20^ (US)	2007	758	0.5%	2.5%	12.9%
Yap^8^ (Singapore)	2010	50	6%	4%	10%
Nelson^15^ (US)	2010	102	1%	1%	6%
Selber^16^ (US)	2010	97	1%	0%	2%
Enajat^17^ (Sweden)	2010	18	0%	0%	6%
NHS^22^ (UK – all free flaps)	2009	974	2%	2.5%	–
Present study	2011	159	0%	9%	9%

There was no total flap loss although there were 14 partial flap losses. (We define partial flap loss as any area of skin and flap tissue that is necrotic and needs surgical debridement.) Early on in the series the shape of the planned flap was de-epithelialised on the abdomen before transfer to the chest, which, on occasion, led to significant tension in the flap. This tension was felt to be responsible for some of the partial flap necrosis. This resolved almost completely by leaving the shaping until the flap had been transferred to the chest.

Adequate post-operative pain control has been shown to improve outcomes in many different types of surgery and is critical in DIEP breast reconstruction patients.^13^ Our peri-operative analgesia regimen includes an intrapleural block performed at induction of anaesthesia, a rectus sheath block during wound closure and morphine PCA post-operatively, which is converted to oral analgesics when possible. When we compare the total volumes of morphine used in our population with a population without the intrapleural block from the literature, we note that our morphine consumption is less and therefore our post-operative pain appears to be less (28mg [SD: 36mg] vs 70mg [SD: 50mg]).^14^

## Conclusions

In our experience, DIEP breast reconstruction is a significant and complex operation that is demanding of both the patient and the surgeon but can give a superb cosmetic result in shape, warmth and movement that is very difficult to reproduce using any other reconstructive technique. We have demonstrated a definite leaning curve with the acquisition of the technical aspects of the operation and the provision of peri-operative venous thromboembolic prophylaxis and analgesia, which is reflected in the decline in complications throughout our series. Ongoing audit of our outcomes and subsequent refinement of the care pathway we offer to patients has allowed us to significantly reduce morbidity associated with the procedure.
